# Acyl Chain-Dependent Effect of Lysophosphatidylcholine on Endothelium-Dependent Vasorelaxation

**DOI:** 10.1371/journal.pone.0065155

**Published:** 2013-05-31

**Authors:** Shailaja P. Rao, Monika Riederer, Margarete Lechleitner, Martin Hermansson, Gernot Desoye, Seth Hallström, Wolfgang F. Graier, Saša Frank

**Affiliations:** 1 Institute of Molecular Biology and Biochemistry, Center of Molecular Medicine, Medical University Graz, Graz, Austria; 2 Department of Biochemistry, Institute of Biomedicine, University of Helsinki, Helsinki, Finland; 3 Clinic of Obstetrics and Gynecology, Medical University Graz, Graz, Austria; 4 Institute of Physiological Chemistry, Center of Physiological Medicine, Medical University Graz, Graz, Austria; 5 University of Applied Sciences, Biomedical Science, Graz, Austria; Fundação Oswaldo Cruz, Brazil

## Abstract

Previously we identified palmitoyl-, oleoyl-, linoleoyl-, and arachidonoyl-lysophosphatidylcholine (LPC 16:0, 18:1, 18:2 and 20:4) as the most prominent LPC species generated by endothelial lipase (EL). In the present study, we examined the impact of those LPC on acetylcholine (ACh)- induced vascular relaxation. All tested LPC attenuated ACh-induced relaxation, measured ex vivo, using mouse aortic rings and wire myography. The rank order of potency was as follows: 18:2>20:4>16:0>18:1. The attenuating effect of LPC 16:0 on relaxation was augmented by indomethacin-mediated cyclooxygenase (COX)-inhibition and CAY10441, a prostacyclin (PGI_2_)- receptor (IP) antagonist. Relaxation attenuated by LPC 20:4 and 18:2 was improved by indomethacin and SQ29548, a thromboxane A_2_ (TXA_2_)- receptor antagonist. The effect of LPC 20:4 could also be improved by TXA_2_- and PGI_2_-synthase inhibitors. As determined by EIA assays, the tested LPC promoted secretion of PGI_2_, TXA_2_, PGF_2α_, and PGE_2_, however, with markedly different potencies. LPC 16:0 was the most potent inducer of superoxide anion production by mouse aortic rings, followed by LPC 18:2, 20:4 and 18:1, respectively. The strong antioxidant tempol recovered relaxation impairment caused by LPC 18:2, 18:1 and 20:4, but not by LPC 16:0. The tested LPC attenuate ACh-induced relaxation through induction of proconstricting prostanoids and superoxide anions. The potency of attenuating relaxation and the relative contribution of underlying mechanisms are strongly related to LPC acyl-chain length and degree of saturation.

## Introduction

Lysophosphatidylcholines (LPC) are bioactive phospholipids generated by various biological processes including: i) phospholipase A_2_ (PLA_2_)-catalysed cleavage of plasma membrane and lipoprotein phosphatidylcholine (PC) [1], ii) lecithin cholesterol acyltransferase (LCAT) activity in high density lipoproteins (HDL) [2], and iii) oxidation of low density lipoproteins (LDL) [3]. In contrast to exclusively saturated LPC species generated by aforementioned processes, both hepatic lipase (HL) and endothelial lipase (EL) generate in addition to LPC 16:0 unsaturated species 18:1, 18:2 and 20:4 by cleaving HDL-PC [4], [5].

Under physiological conditions the plasma concentration of LPC is around 100–170 µM [6], with elevations under pathophysiological conditions up to millimolar concentrations in e.g. hyperlipidemic subjects [7]. LPC in plasma are distributed between albumin and other carrier proteins as well as lipoproteins [8], [9]. Free LPC might occur locally during an excessive lipolysis and concomitant saturation of carrier proteins with lipolysis products. The interaction of free LPC with vascular endothelium, as found for LPC 16:0, results in altered endothelial function and impaired vascular reactivity [10], [11].

The maintenance of normal vascular tone is largely dependent on the capability of vascular endothelium to maintain the fine balance between endothelium-derived relaxing factors (EDRF) and endothelium-derived contracting factors (EDCF). Upon stimulation with various agonists EDRF and EDCF released from vascular endothelial cells diffuse to underlying smooth muscle cells, where they act on specific receptors and cause relaxation or contraction [12]. While nitric oxide (NO) and endothelium-derived hyperpolarizing factor (EDHF) are principal EDRF, prostanoids, the products of cyclooxygenase (COX)-1 and -2, may act as both EDRF and EDCF [12], [13].

Endothelium-derived PGI_2_ promotes relaxation of underlying vascular smooth muscle cells through activation of PGI_2_- (IP) receptors [13]. However, under certain conditions, PGI_2_, like TXA_2_, causes contraction through activation of TXA_2_- (TP) receptors [14]. PGE_2_ promotes relaxation via PGE_2_- (EP) receptors subtypes EP_2_ and EP_4_, whereas by acting via EP_1_, EP_3_ and TP receptors it causes constriction [15], [16]. Likewise, PGF_2α_ and isoprostanes, cause contraction through activation of TP receptors on vascular smooth muscle cells [17], [18]. Additionally, endothelium-derived ROS may act as potent EDCF either directly by promoting depolarization of vascular smooth muscle [19] or indirectly by reducing NO bioavailability [20].

Previously we found that the capacity and underlying mechanisms of palmitoyl-LPC (16:0 LPC), oleoyl-LPC (18:1 LPC), linoleoyl-LPC (18:2 LPC) and arachidonoyl-LPC (20:4 LPC) to modulate endothelial prostanoid production were remarkably different and related to the acyl-chain length as well as degree of saturation [21]. At present only the impact of 16:0 LPC on vascular reactivity has been investigated.

Here we tested the hypothesis of acyl chain dependency of LPC in altering vascular reactivity. To this end we compared the effects of LPC 18:1, 18:2 and 20:4 with LPC 16:0 on ACh-induced vasorelaxation in an *ex vivo* system using mouse aortic rings and myography. We found that the tested LPC attenuate ACh-induced relaxation through induction of proconstricting prostanoids and superoxide anions whereby the potency of attenuating relaxation and the relative contribution of underlying mechanisms are strongly related to LPC acyl-chain length and degree of saturation.

## Materials and Methods

### LPC

LPC 16:0, 18:1, 18:2 and 20:4 were purchased from Avanti Polar Lipids, Alabaster, AL or prepared as described [6]. LPC were dissolved in chloroform/methanol and stored at −20°C under argon atmosphere. Required amounts of LPC were dried under a stream of nitrogen or argon and re-dissolved in PBS (pH 7.4) before the experiment.

### Mice and tissue preparation

Mice received care in accordance with the Austrian law on experimentation with laboratory animals, which is based on the U.S. National Institutes of Health guidelines. Male C57BL/6 mice (10–12 weeks old) provided by Himberg, Austria, were killed by cervical dislocation. The descending thoracic aorta was isolated and dissected free of adherent tissue.

### Organ Chamber Experiments

Aortic rings approximately 2 mm in length were cut from descending thoracic aorta. The arterial rings were positioned in small wire myograph chambers (Danish MyoTechnology, Aarhus, Denmark), which contained physiological salt solution (PSS) (114 mM NaCl, 4.7 mM KCl, 0.8 mM KH_2_PO_4_, 1.2 mM MgCl_2_, 2.5 mM CaCl_2_, 25 mM NaHCO_3_ and 11 mM D-glucose pH 7.4) aerated with 5% CO_2_/95% O_2_ at 37°C. The myograph chambers were connected to force transducers for isometric tension recording (PowerLab, ADInstruments). The rings were heated in PSS buffer to 37°C. An initial preload of 10 mN was applied, and the rings were allowed to stabilize for 30 min. PSS containing 60 mM KCl was used to determine maximum contractility of the tissue. When the developed tension attained its peak value, the rings were relaxed by rinsing with the buffer. Next, the rings were pre-contracted with increasing concentrations of norepinephrine (NE) (1 nM–0.3 µM) (Sigma-Aldrich) to produce 80% of the maximum contraction achieved by 60 mM KCl, followed by endothelium-dependent relaxation to cumulatively increasing concentrations of acetylcholine chloride (ACh) (1 nM–0.3 µM) (Sigma-Aldrich). After washout and equilibration, the rings were preincubated with 10 µM LPC in the presence or absence of inhibitors for 30 minutes, followed by contraction (NE) – relaxation (ACh) cycle as described above. Relaxation values were expressed as a percentage of the NE-induced contraction. The endothelium-independent relaxation was examined by exposure of rings to increasing concentrations (0.1 nM to 30 nM) of sodium nitroprusside (SNP) (Sigma-Aldrich), a nitric oxide (NO)-donor.

### Pharmacological inhibitors

Indomethacin (non-selective COX inhibitor; 20 µM), N5-[imino(nitroamino)methyl]-L-ornithine (L-NNA; eNOS inhibitor; 200 µM), Tempol (superoxide dismutase (SOD)- mimetic; 200 µM), SQ29548 (TXA_2_ receptor antagonist; 10 µM), Diethyldithiocarbamic acid diethylammonium salt (DETCA) (SOD inhibitor; 10 µM) and TIRON (superoxide ion scavenger; 100 µM) were from Sigma (Saint Louis, MO). Tranylcypromine (PGI_2_ synthase inhibitor; 10 µM) was from Calbiochem. Furegrelate (TXA_2_ synthase inhibitor; 10 µM) and CAY10441 (IP receptor antagonist; 1 µM) were from Cayman Chemicals (Ann Arbor, Michigan, USA). Stock solutions (10 mM) of Indomethacin and Tranylcypromine were made in dimethylsulfoxide (DMSO) and further diluted with distilled water. The stock solution (10 mM) of SQ29548 was prepared in ethanol and further diluted with distilled water. All other drugs were dissolved in distilled water.

### Prostanoid measurement

Mouse aortic rings (approximately 2-mm in length) were incubated in 200 µl aerated PSS under cell culture conditions at 37°C for 1 h. Thereafter, buffer was replaced with fresh PSS supplemented with PSS (control) or LPC (10 µM) followed by further incubation under cell culture conditions at 37°C for 1 h. The buffers were flash frozen in liquid nitrogen for subsequent prostanoid quantification and rings were homogenized for protein quantification. Protein concentration was determined with the BCA protein assay kit (Novagen, Darmstadt, Germany). The concentrations of 6-Keto-PGF_1α_, TXB_2_, PGE_2_ and PGF_2α_ were measured by corresponding correlate-EIA kits (Cayman, Ann Arbor, MI) according to the manufacturer's protocol.

### Superoxide anions measurement

Superoxide anions were measured as described [22] with some modifications. Mouse aortic rings were equilibrated in 100 µl PSS buffer containing 10 µM DETCA, an SOD-inhibitor and 10 µM lucigenin (Sigma) at 37°C for 30 minutes. LPC (10 µM) was added to the tubes immediately before measurements. The luminometer (Lumat LB9501, Berthold technologies, Germany) was set up to report arbitrary units of emitted light (RLU). Measurements were taken in triplicates every 10 seconds. In addition, blank measurements with and without aortic rings were collected in the same way to subtract background emission. The amounts of released superoxide anions (chemiluminiscence units) were normalised to protein content of respective aortic rings. The RLU obtained in control incubations with PSS were set to 100% and the RLU obtained by LPC's were expressed as percentage of the control

### Nitrite determination

Nitrite as an indicator of NO production was determined according to a previously described fluorometric HPLC method [23] utilizing the reaction of nitrite with 2,3-diaminonaphthalene (DAN). In brief, the nitrite levels were determined in the myography incubation buffers. Samples (500 µl) were taken and snap frozen in liquid nitrogen. After thawing 100 µL of the sample (incubation buffer) was incubated at 24°C with 10 µL of 316 µmol/L DAN (in 0.62 mol/L HCl) for 10 min, followed by addition of 10 µL of 2.8 mol/L NaOH. This reaction mixture was directly used for chromatographic separation (injection volume: 20 µL) of the formed 2, 3-naphthotriazole (NAT). Nitrite standards (range: 0–2 µmol/L) were derivatized accordingly. NAT was isocratically separated on a 5-µm ODS hypersil column (150×4.6 mm) guarded by a 5-µm ODS hypersil column (10×4.6 mm; Uniguard holder) with a 30 mmol/L sodium phosphate buffer (pH 7.5) containing 50% methanol (flow rate: 0.8 mL/min). Fluorescence was monitored at an excitation wavelength of 375 nm and an emission wavelength of 415 nm. The HPLC apparatus consisted of an L-2200 autosampler, L-2130 HTA pump and L-2480 fluorescence detector (VWR Hitachi, Tokyo, Japan). Detector signals were recorded with a personal computer. The EZchrom Elite (Scientific Software Inc., San Ramon, CA USA) was used for data requisition and analysis. The detection limit for nitrite was 10 pmol/mL.

### Statistical Analysis

EC_50_ values (the ACh concentrations required to achieve 50% of maximal relaxation) are expressed as mean with 95% confidence intervals. Data are otherwise expressed as mean ± SEM. The significance of the difference between group means was analyzed by two-way analysis of variance and the Bonferroni-post test for samples. For prostanoid, nitrite and ROS measurements control and LPC treated aortic rings were compared by student's T-test. Values of P<0.05 (*), P<0.01 (**), and P<0.001 (***) were taken as statistically significant. Statistical analysis was performed by Prism Version 4.0 (GraphPad Software, USA).

## Results

### LPC attenuate ACh-induced endothelium-dependent relaxation

**Figure 1:**
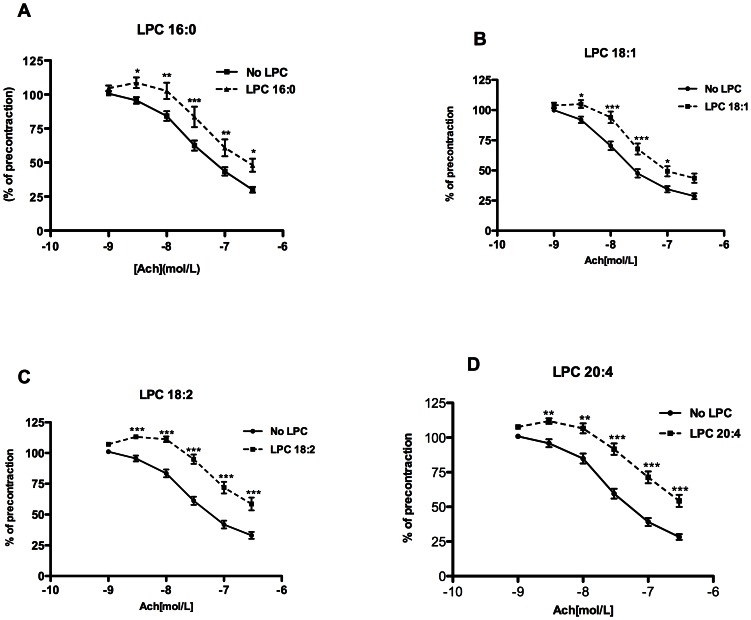
ACh-induced relaxation of mouse aortic rings is attenuated by LPC. The rings were preincubated without (no LPC) or with 10 µM LPC 16:0 (A), 18:1 (B), 18:2 (C) or 20:4 (D) for 30 minutes, followed by precontraction with NE and cumulative addition of ACh. Relaxation values were expressed as a percentage of the NE-induced contraction. Results of each experimental condition are mean ± SEM of 24 rings for each case from 6 mice. *P<0.05, **P<0.01***P<0.001.

All tested LPC attenuated aortic ring relaxation to cumulatively increasing concentrations of ACh with rank order of potency as follows: 18:2>20:4>16:0>18:1 (Fig. 1). EC_50_ values for LPC 18:2, 20:4, 16:0 and 18:1 were 354 nM (261–479), 298 nM (222–401), 214 nM (143–314) and 115 nM (86.2–155), respectively. The subsequent relaxation of the same rings to SNP, following precontraction with NE, was not affected by either of the tested LPC (Fig. S1), indicating that responsiveness of aortic smooth muscle layers to NO was not impaired by prior exposure to LPC.

### The role of COX and PGI_2_ in LPC- mediated attenuation of vascular relaxation

**Figure 2:**
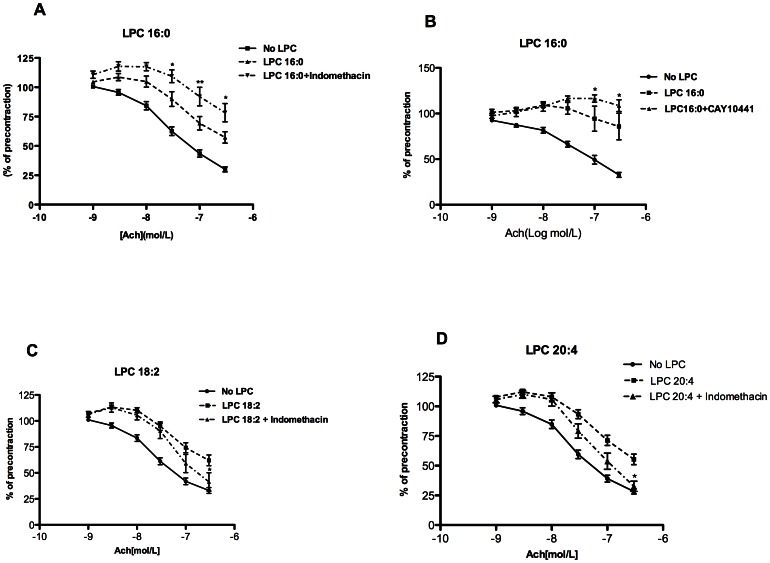
Impact of COX-inhibition and prostacyclin-action on LPC-induced attenuation of relaxation. The rings were preincubated without (no LPC) or with 10 µM of indicated LPC in the absence or presence of (A,C,D) indomethacin, a non-selective COX inhibitor (20 µM) or (B) CAY10441, a IP receptor antagonist (1 µM) for 30 minutes, followed by precontraction with NE and cumulative addition of ACh. Relaxation values were expressed as a percentage of the NE-induced contraction. Indomethacin improved relaxation attenuated by LPC 18:2 (C) and 20:4 (D). Relaxation attenuated by LPC 16:0 was exaggerated by indomethacin and CAY10441. Results are mean ± SEM of 20 rings for each case from 10 mice (A), 8 rings for each case from 3 mice (B) and 12 rings for each case from 6 mice (C,D).

To examine whether COX-derived vasoconstricting prostanoids are responsible for the observed LPC-induced attenuation of relaxation, myography experiments were performed in the absence or presence of the non-selective COX inhibitor, indomethacin. In contrast to our expectation, indomethacin augmented the attenuating effect of LPC 16:0 on ACh-induced relaxation (Fig. 2A). Similar finding was obtained with CAY10441, an IP receptor antagonist (Fig. 2B). In contrast, indomethacin improved the relaxation attenuated by LPC 18:2 (Fig. 2C) and 20:4 (Fig. 2D), but had no effect on the attenuation caused by LPC 18:1 (Fig. S2).

### TP receptors are involved in LPC 18:2- and 20:4- mediated attenuation of vascular relaxation

**Figure 3:**
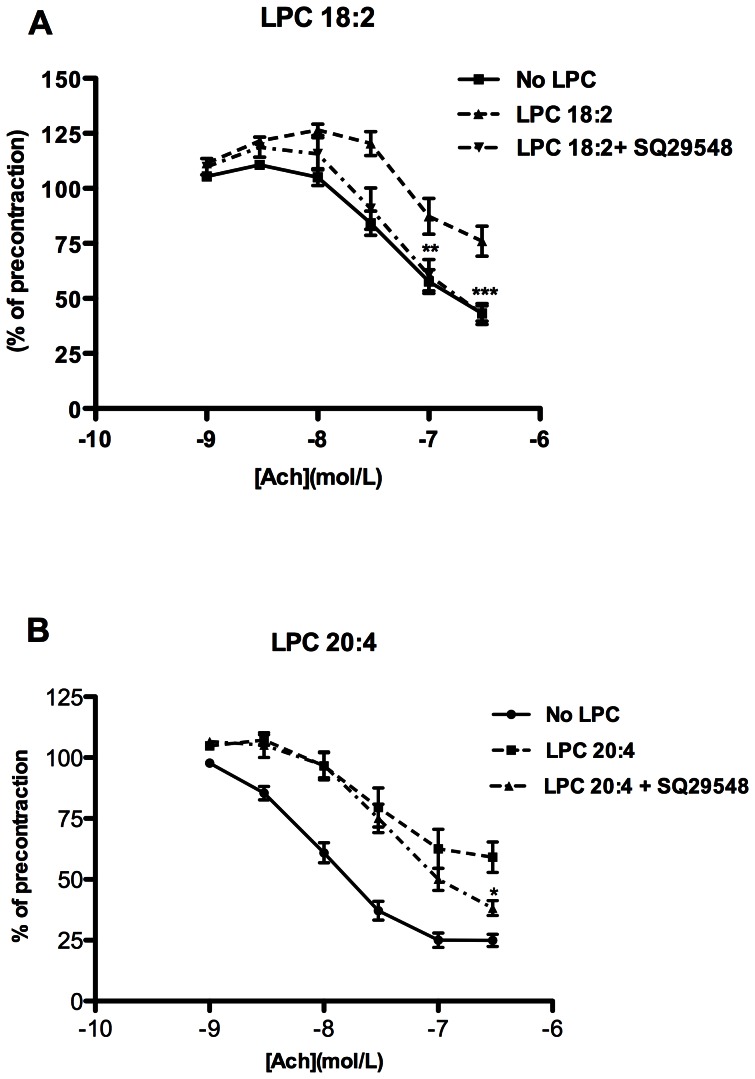
Blocking of TP receptor improves LPC 18:2- and 20:4-induced attenuation of relaxation. The rings were preincubated without (no LPC) or with 10 µM LPC 18:2 (A) or 20:4 (B) in the absence or presence of 10 µM SQ29548, a TP receptor antagonist for 30 minutes, followed by precontraction with NE and cumulative addition of ACh. Results are mean ± SEM of 12 rings from 6 mice. *P<0.05, **P<0.01***P<0.001.

Considering the well-established importance of TP receptors in mediating endothelium-dependent contractions [24], [25], we examined whether SQ29548, a TP receptor antagonist could attenuate the inhibitory effect of LPC on vasorelaxation. SQ29548 markedly attenuated the inhibitory effect of LPC 18:2 (Fig. 3A) and significantly improved relaxation attenuated with LPC 20:4 (Fig. 3B), but had no significant impact on relaxation attenuated with LPC 16:0 or 18:1, respectively (not shown).

### The role of TXA_2_ and PGI_2_ in LPC 18:2- and 20:4- mediated attenuation of vascular relaxation

**Figure 4:**
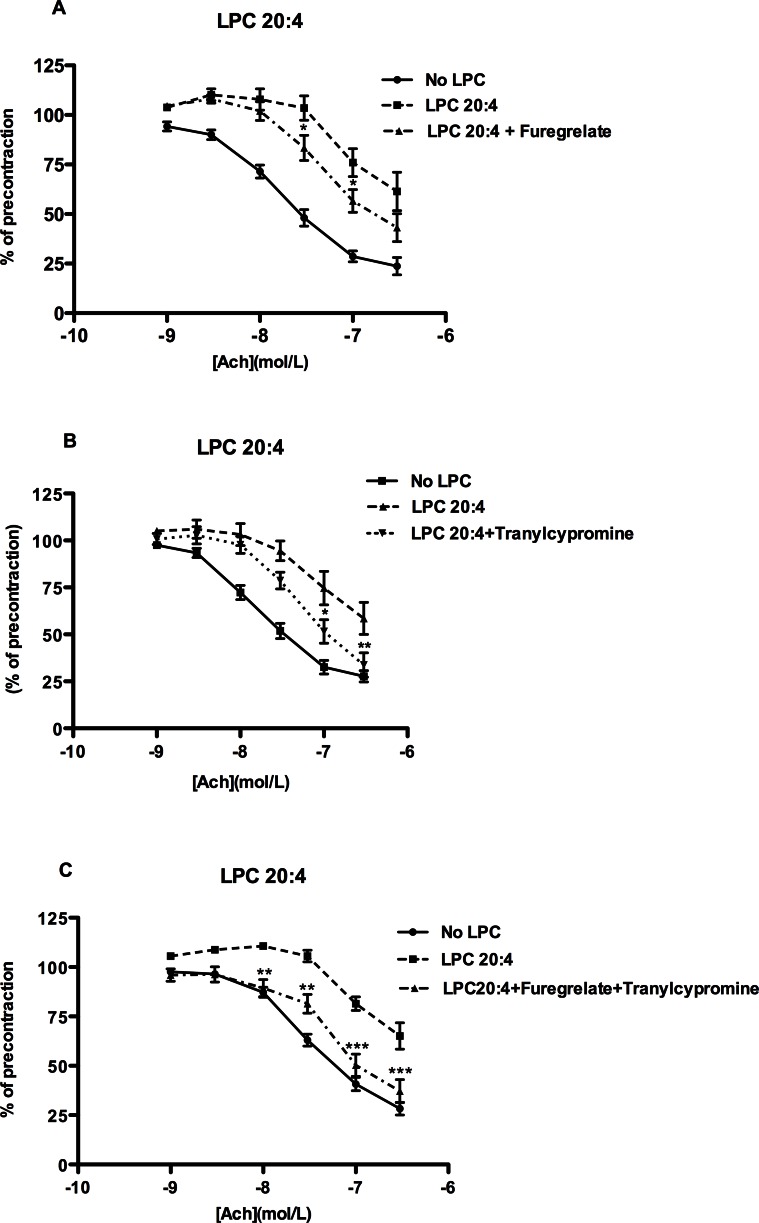
Inhibition of TXA_2_- and PGI_2_- synthase improves relaxation attenuated by LPC 20:4. The rings were preincubated without (no LPC) or with LPC 20:4 in the absence or presence of 10 µM furegrelate, a TXA_2_ synthase inhibitor (A) or 10 µM tranylcypromine, a PGI_2_ synthase inhibitor (B) or a combination of both (C) for 30 minutes, followed by precontraction with NE and cumulative addition of ACh. Results are mean ± SEM of 12 rings from 6 mice. *P<0.05, **P<0.01.

Since TP receptors can be activated by both TXA_2_ and PGI_2_ [17], we tested the involvement of both prostanoids in LPC 18:2- and 20:4-induced attenuation of relaxation. Attenuated relaxation observed in the presence of LPC 20:4 was markedly improved upon inhibition of TXA_2_-synthase by furegrelate (Fig. 4A) as well as upon inhibition of PGI_2_-synthase by tranylcypromine (Fig. 4B). The co-application of both inhibitors resulted in further improvement of relaxation, however without complete restoration of relaxation, suggesting the involvement of additional 20:4 LPC-induced vasoconstricting factors. Neither of both inhibitors could recover relaxation impaired by LPC 18:2 (Fig. S3A, S3B).

### Prostanoid release from LPC-treated aortic rings

**Figure 5:**
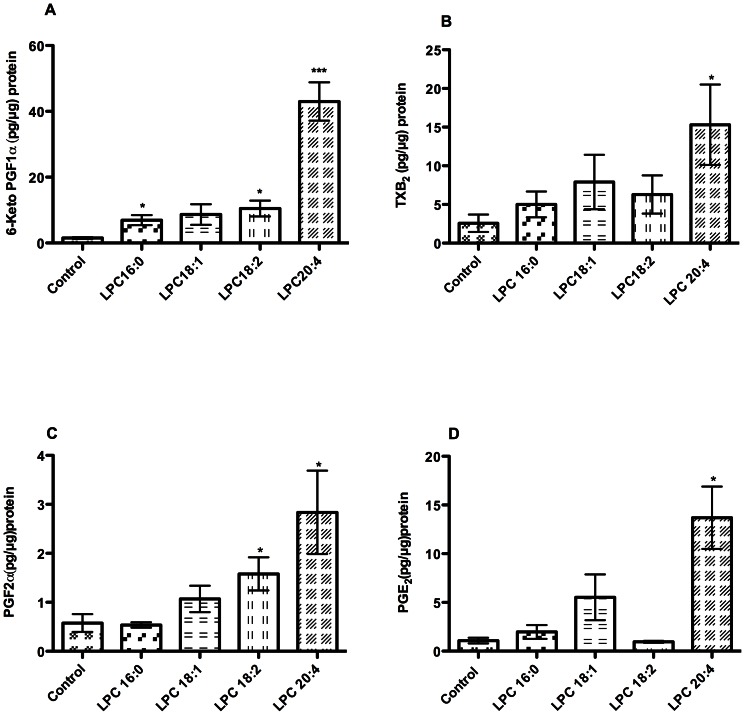
Prostanoid release from LPC-treated aortic rings. The rings were incubated in 200 µl aerated PSS under cell culture conditions at 37°C for 1 h. Thereafter, buffer was replaced with fresh PSS supplemented with PSS (control) or 10 µM LPC followed by further incubation under cell culture conditions at 37°C for 1 h. (A) 6-keto PGF_1α_, a stable degradation product of PGI_2_ (B), TXB_2_, a stable degradation product of TXA_2_, (C) PGE_2_ and (D) PGF_2α_ were quantified by EIA assays and rings were solubilized for protein quantification. Results shown in A and B are means ± SD of four experiments and those in C and D of three experiments, done in triplicates. *P<0.05, **P<0.01***P<0.001.

To examine whether the production of prostanoids implicated in LPC-induced attenuation of relaxation (Figs. 1–4) was increased by LPC, we measured concentrations of prostanoids produced and secreted by aortic rings upon incubation with LPC. Compared with PSS-treated control incubations, LPC 20:4 was the most potent inducer of PGI_2_ production (measured as 6-Keto PGF_1α_, a stable degradation product of PGI_2_), followed by 18:2 and 16:0 (Fig. 5A). The effect of LPC 18:1 concerning prostanoids did not reach statistical significance (Fig. 5A). The release of TXB_2_ was significantly increased only with LPC 20:4 (Fig. 5B). While PGE_2_ production was significantly increased only upon incubation with LPC 20:4 (Fig. 5C), the levels of PGF_2α_ were significantly increased with LPC 20:4 and 18:2, respectively (Fig. 5D).

### LPC-induced oxidative stress contributes to LPC-induced attenuation of vascular relaxation

**Figure 6:**
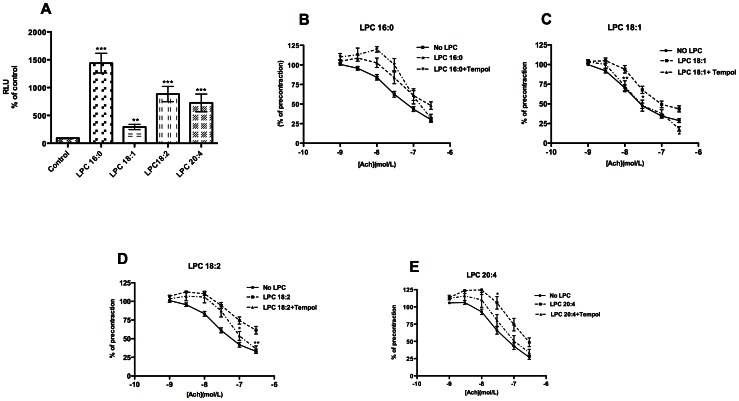
ROS are induced by LPC and contribute to LPC-induced impairment of relaxation. A) The rings were equilibrated in 100 µl PSS buffer containing 10 µM DETCA and 10 µM lucigenin at 37°C for 30 minutes, followed by addition of PSS (control) or LPC (10 µM). Emitted light (RLU) was recorded every 10 seconds for 30 seconds. The RLU were normalised to protein content of respective aortic rings and expressed as percentage of control set to 100%. Results are means ± SEM of three separate experiments, each performed with three rings for each LPC. The rings were preincubated without (no LPC) or with 10 µM LPC 18:1 (B), 18:2 (C), 20:4 (D) or 16:0 (E) in the absence or presence of 200 µM Tempol for 30 minutes, followed by precontraction with NE and cumulative addition of ACh. Results for each condition are mean ± SEM of 12 rings from 6 mice. *P<0.05, **P<0.01.

Since superoxide anions are established EDCF [17], we examined whether their production is triggered by LPC and whether they contribute to the observed LPC-induced impairment of relaxation. As shown in Figure 6A all LPC induced superoxide anion production in mouse aortic rings with the following order of potency: 16:0>18:2>20:4>18:1. The SOD mimetic tempol [26] improved relaxation impairment caused by LPC 18:1 (Fig. 6C), 18:2 (Fig. 6D) and 20:4 (Fig. 6E) but not that caused by LPC 16:0 (Fig. 6B).

## Discussion

The present study investigated the effect of LPC 16:0, 18:1,18:2 and 20:4, the most prominent LPC in human plasma [6], on endothelium-dependent relaxation in response to ACh. Previously, we identified these LPC as major hydrolysis products generated by the action of EL on HDL [4]. Considering the very high plasma levels under pathophysiological conditions (e.g. hyperlipidemic subjects) and their production by EL on the surface of vascular endothelial cells, these LPC might have a pronounced effect on endothelial function and vascular reactivity. Numerous studies have examined the effect of LPC on vascular reactivity. However, in these studies exclusively LPC 16:0 was used as a model LPC. To the best of our knowledge the present study is the first one to address the effect of unsaturated LPC 18:1, 18:2 and 20:4 on ACh-induced relaxation and to compare the effect with the saturated LPC 16:0.

We found that all LPC caused a pronounced attenuation of endothelium-dependent relaxation (EDR) to ACh with remarkable acyl-chain dependent differences regarding the potency and underlying mechanisms. LPC did not alter the SNP-induced relaxation (Fig. S1). This demonstrates that LPC are not toxic to smooth muscle cells and that the observed LPC-mediated attenuation of relaxation is an endothelium-dependent effect. Neither of the tested LPC modified the contractile response to NE, nor did they induce contraction when co-applied with the eNOS inhibitor L-NNA (not shown).

L-NNA completely inhibited, whereas indomethacin had no effect on ACh-induced relaxation of mouse aortic rings (Fig. S4A, S4B). Thus, NO seems to be the major mediator of ACh-induced relaxation in our experimental model. This is in line with the inability of ACh to alter vascular tone in aortic rings from eNOS deficient mice [27]. Accordingly, the observed attenuation of relaxation caused by LPC may reflect increased production and activity of EDCF, with concomitant counteraction of ACh-induced NO-mediated relaxation. This is similar to the inability of endothelial NO to curtail the effect of EDCF observed in arteries of aging and diseased (essential hypertension, diabetes) animals and humans [28]–[30]. In spontaneous hypertensive rats (SHR) and in essential hypertensive patients, impaired vasodilatation was almost normalized by the COX-inhibitor indomethacin, indicating that COX-derived vasoconstrictors are key EDCF responsible for impaired endothelial function and blunted vasorelaxation [30].

Improvement of relaxation attenuated by LPC 18:2 and 20:4 upon inhibition of both COX (Fig. 2C, 2D) and TP receptors (Fig. 3A, 3B), indicated the involvement of COX-derived EDCF, which are capable of inducing contraction by acting via TP receptors [17], [30]. These receptors are highly expressed in mouse aortic smooth muscle cells [31]. Both PGI_2_ and TXA_2_ were markedly induced by LPC 20:4 (Fig. 5A, 5B). These prostanoids are capable of activating TP receptors [17] and may, hence, have a major contribution to the LPC 20:4-effect (Fig. 4A–C). A recent study clearly showed that in mouse aorta both exogenously applied PGI_2_ (0.03 µM) as well as endogenous, ACh-induced PGI_2_ potently induced vasoconstriction by acting on TP receptor [32]. In that study, the concentration of 6-keto PGF_1_α upon ACh-stimulation was 2 pg/µg tissue, which is 20 times less than what we observed upon exposure of rings to 20:4 LPC (Fig. 5A). Accordingly, 40 pg/µg of 6-keto PGF_1_α in 20:4 LPC-treated rings strongly argues for a PGI_2_-induced vasoconstriction via TP receptors in our experimental model. Additionally, the decreased ability of IP receptors to promote relaxation, as found in vascular smooth muscles of SHR [30], and/or markedly higher levels of TP compared with IP receptors in mouse aortic smooth muscle cells [31], might facilitate vasoconstriction in 20:4 LPC-treated rings, despite increased PGI_2_ production. Future experiments should reveal whether responsiveness of IP receptors to PGI_2_ or its stable analogue, iloprost, is altered by LPC.

Neither TXA_2_ nor PGI_2_ were involved in LPC 18:2-induced relaxation attenuation (Fig. S3A, S3B). However, the robust counteracting effect of the TP receptor antagonist on the LPC 18:2-induced relaxation attenuation (Fig. 3A) strongly suggests the existence and action of some LPC 18:2-induced TP-receptor agonists. Because LPC 18:2 induced PGF_2α_ (Fig. 5D) which can activate TP receptors [17], the LPC 18:2-induced attenuation of relaxation might at least in part be due to the EDCF-activity of induced PGF_2α_. Furthermore, isoprostanes such as 8-epi-PGF_2α_ formed non-enzymatically by ROS induced peroxidation of cell membrane polyunsaturated fatty acids, as well as PGH_2_, a direct product of COX, might by acting via TP receptors, contribute to observed attenuating effect of LPC 18:2 on relaxation. Likewise, PGE_2_ and PGF_2α_ [24] both markedly increased with LPC 20:4 (Fig. 5C, 5D) might contribute by acting via the TP receptors, to the relaxation attenuation induced by LPC 20:4. Due to the negligible expression of PGE_2_ receptors EP1 and EP3 (receptors associated with contraction) in mouse aortic smooth muscle cells, their contribution to contraction seems unlikely [27].

Previous studies found contradictory effects of LPC 16:0 on vasorelaxation, most probably attributable to vascular bed- and species- specific differences in tissue responsiveness to LPC [33]–[36]. In our experimental system LPC 16:0 increased the nitrite levels (indicative of NO levels) in organ bath of aortic rings exposed to ACh (Fig. S5), arguing against decreased NO as a cause of relaxation impairment induced by this LPC. Importantly, SNP-induced relaxation was not altered by the presence of LPC 16:0, indicating that responsiveness of smooth muscle cells to NO was not impaired by this LPC (Fig. S1A).

We found that in contrast to the tested unsaturated LPC species, the rate of ACh-induced relaxation in the presence of LPC 16:0 is the sum of 16:0-LPC-induced attenuation of relaxation (by a so far unknown mechanism) and promotion of relaxation by 16:0-LPC-induced PGI_2_ (Figs. 2B and 5A). By contrast, a similar induction of PGI_2_ by LPC 18:2 (Fig. 5A) failed to promote relaxation. This difference between LPC 16:0 and 18:2 is not clear, but one can speculate it might be due to the fact that PGI_2_ is the only prostanoid upregulated by LPC 16:0, whereas the action of LPC 18:2-induced PGI_2_ might be disturbed by concomitantly upregulated PGF_2α_ or by detrimental effect of LPC 18:2 on IP receptor functionality.

Considering the short exposure of aortic rings to LPC (45 min) in our experiments, the observed augmenting effect of LPC 16:0 on prostanoid production may not reflect upregulation of underlying enzymes such as COX-2 or respective prostanoid synthases. Indeed, in our recent study, the LPC-induced COX-2 protein upregulation in endothelial cells was detectable not earlier than after 3 h of incubation with LPC [37]. Hence, in line with our previous findings [21] the observed increase in prostanoid production upon exposure of aortic rings to LPC is rather a consequence of an acute effect of LPC, namely LPC-induced increase in cytosolic calcium concentration with concomitant activation of phospholipase-mediated release of arachidonic acid from membrane phospholipids. Because in contrast to LPC 16:0, 18:1 and 18:2, LPC 20:4 not only induces arachidonic acid release, but also provides its own arachidonic acid to COX [21], 20:4 LPC elicited the highest prostanoid production in aortic rings (Fig. 5A).

Besides vasoconstricting prostanoids, ROS are established EDCF [17]. In contrast to LPC 18:1, 18:2 and 20:4, LPC 16:0-induced attenuation of relaxation could not be improved by tempol, a SOD mimetic [26] (Fig. 6). Similarly, LPC 16:0 promoted ROS production in rat aortic rings, but MnCl_2_, another SOD mimetic, failed to restore the impaired relaxation [36]. As found for tempol, a combination of 10 µM DETCA (SOD inhibitor) and 100 µM TIRON (ROS scavenger) failed to improve relaxation attenuated by LPC 16:0 (Fig. S6). The most prominent tempol-mediated improvement of relaxation was observed with LPC 18:1, most likely due to the fact that only increased ROS and not concomitantly increased vasoconstricting prostanoids underlie the LPC 18:1-induced relaxation attenuation.

Since LPC used in the present study were prepared by PLA_2_-mediated cleavage of di-16:0-, -18:1-, -18:2- and -20:4-PC [6], applied LPCs are *sn-1*-acyl *sn-2*-lyso LPC. This is in a good accordance with situation *in vivo* where *sn-1*-lyso *sn-2* –acyl LPC generated by *sn-1* phospholipases (such as EL or HL) give rise to *sn-1*-acyl isomers due to a rapid migration of acyl chains (in aqueous medium at neutral pH at 37°C) from the *sn-2* to the deacylated *sn-1* position to give a more stable intermediate [38]. Whether *sn-1* and *sn-2* LPC isomers differ in their biological activities regarding modulation of endothelial function and vascular reactivity remains to be determined.

Based on our results, LPC 16:0, 18:1, 18:2 and 20:4 emerge as important triggers of endothelial dysfunction. The major players responsible for the blunted endothelium-dependent relaxation in aged vessels and in various pathologies (essential hypertension, diabetes and atherosclerosis) are vasoconstricting prostanoids and ROS [17], [28]. The fact that the studied LPC promote the production of these established EDCF strongly argues for the role of LPC 16:0, 18:1, 18:2 and 20:4 as important contributors to endothelial dysfunction in aging and aforementioned pathologies.

Future experiments should reveal the relationship between plasma levels of those LPC and the incidence and degree of endothelial dysfunction in humans and animal models of hypertension.

## Supporting Information

Figure S1 **SNP-induced relaxation is not affected by prior exposure of rings to LPC.** The rings were preincubated without (no LPC) or with 10 µM LPC 16:0 (A), 18:1 (B), 18:2 (C) or 20:4 (D) for 30 minutes, followed by precontraction with NE and cumulative addition of ACh. Rings were rinsed thoroughly with PSS. Thereafter, the rings were precontracted with NE, followed by cumulative addition of SNP (0.1 nM to 30 nM). Relaxation values were expressed as a percentage of the NE-induced contraction. Results of each experimental condition are mean ± SEM of 16 rings for each case from 4 mice.
(TIFF)

Figure S2 **LPC 18:1-induced attenuation of relaxation is not affected by indomethacin.** The rings were preincubated without (no LPC) or with 10 µM LPC 18:1 for 30 minutes, followed by precontraction with NE and cumulative addition of ACh. Relaxation values were expressed as a percentage of the NE-induced contraction. Results are mean ± SEM of 12 rings for each case from 6 mice.
(TIFF)

Figure S3 **Furegrelate and tranylcypromine fail to recover relaxation attenuated by LPC 18:2.** The rings were preincubated without (no LPC) or with LPC 18:2 in the absence or presence of 10 µM furegrelate (A) or 10 µM tranylcypromine (B) for 30 minutes, followed by precontraction with NE and cumulative addition of ACh. Relaxation values were expressed as a percentage of the NE-induced contraction. Results are mean ± SEM of 12 rings for each case from 6 mice.
(TIFF)

Figure S4 **Inhibition of eNOS by L-NNA but not of COX by indomethacin abolishes Ach-induced relaxation in mouse aortic rings.** The rings precontracted with NE were relaxed by a cumulative addition of Ach in the absence (no L-NNA) or presence of 200 µM L-NNA (A) or 20 µM indomethacin (B). Relaxation values were expressed as a percentage of the NE-induced contraction. Results are mean ± SEM of 8 rings for each case from 4 mice.
(TIFF)

Figure S5 **LPC 16:0 increases nitrite levels released from aortic rings exposed to Ach.** The rings were precontracted with NE and relaxed by cumulative addition of Ach. After wash-out of ACh, the same rings were preincubated with 10 µM LPC 16:0 for 30 min followed by a new contraction-relaxation cycle. The nitrite levels were determined in incubation buffers after the first (control) and the second contraction-relaxation cycle (LPC 16:0). Results are mean ± SEM of 16 rings for each case from 4 mice. When LPC was omitted the nitrite levels released from rings were similar in the first and the second contraction-relaxation cycle (not shown).
(TIFF)

Figure S6 **Combination of DETCA (SOD inhibitor) and TIRON (superoxide ion scavenger) fail to counteract LPC 16:0-induced attenuation of relaxation.** The rings were preincubated without (no LPC) or with 10 µM LPC 16:0 in the absence or presence of DETCA (10 µM) and TIRON (100 µM) for 30 minutes, followed by precontraction with NE and cumulative addition of ACh. Results for each condition are mean ± SEM of 8 rings from 4 mice.
(TIFF)
