# Supplementary figures and images for: Acyl Chain-Dependent Effect of Lysophosphatidylcholine on Endothelium-Dependent Vasorelaxation
Source: PLoS One. 2013 May 31;8(5):e65155. doi: 10.1371/journal.pone.0065155 (PMC3669280; doi:10.1371/journal.pone.0065155)

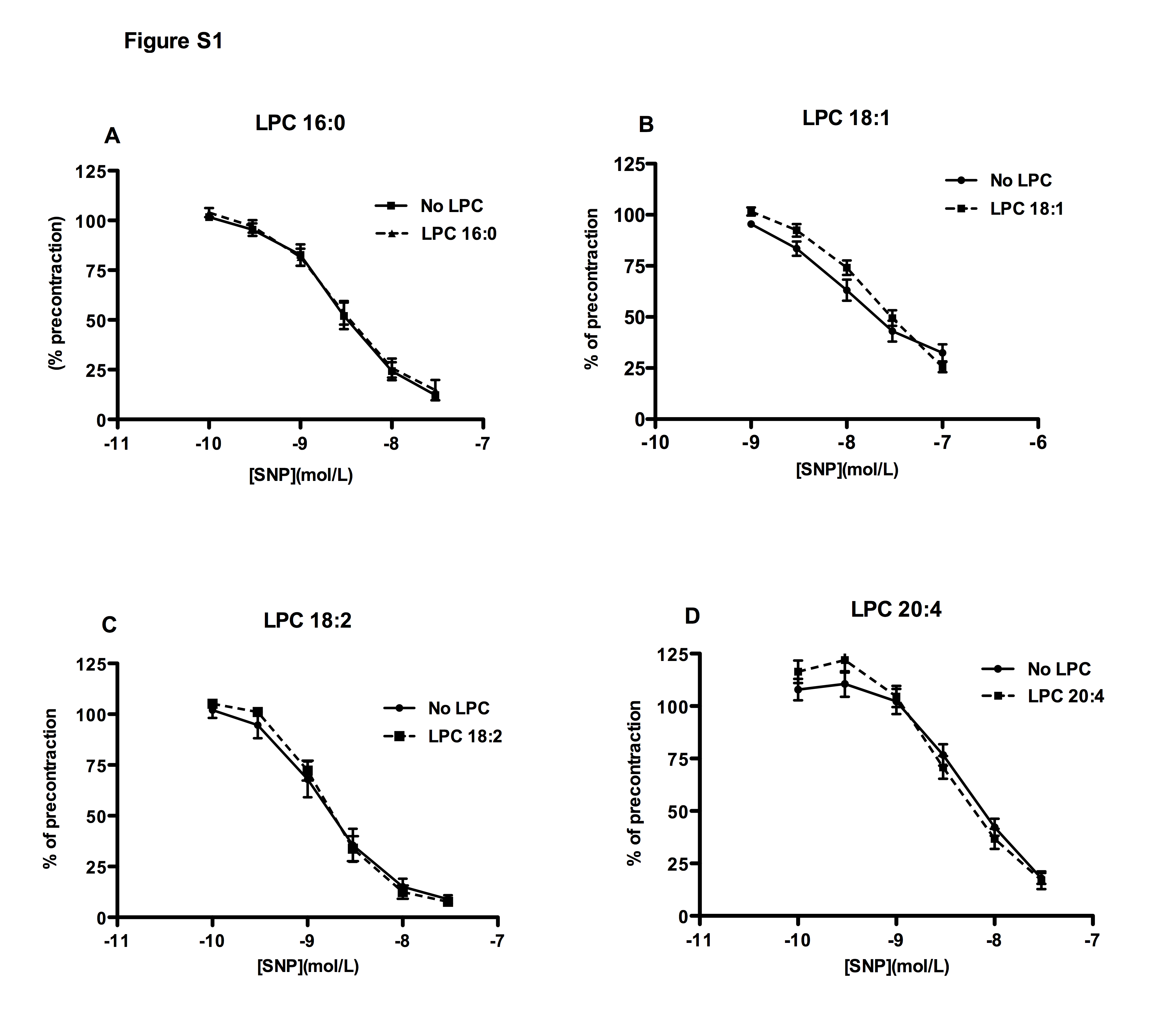

Supplement: Figure S1 — SNP-induced relaxation is not affected by prior exposure of rings to LPC. The rings were preincubated without (no LPC) or with 10 µM LPC 16:0 (A), 18:1 (B), 18:2 (C) or 20:4 (D) for 30 minutes, followed by precontraction with NE and cumulative addition of ACh. Rings were rinsed thoroughly with PSS. Thereafter, the rings were precontracted with NE, followed by cumulative addition of SNP (0.1 nM to 30 nM). Relaxation values were expressed as a percentage of the NE-induced contraction. Results of each experimental condition are mean ± SEM of 16 rings for each case from 4 mice. (TIFF) [file pone.0065155.s001.tiff]

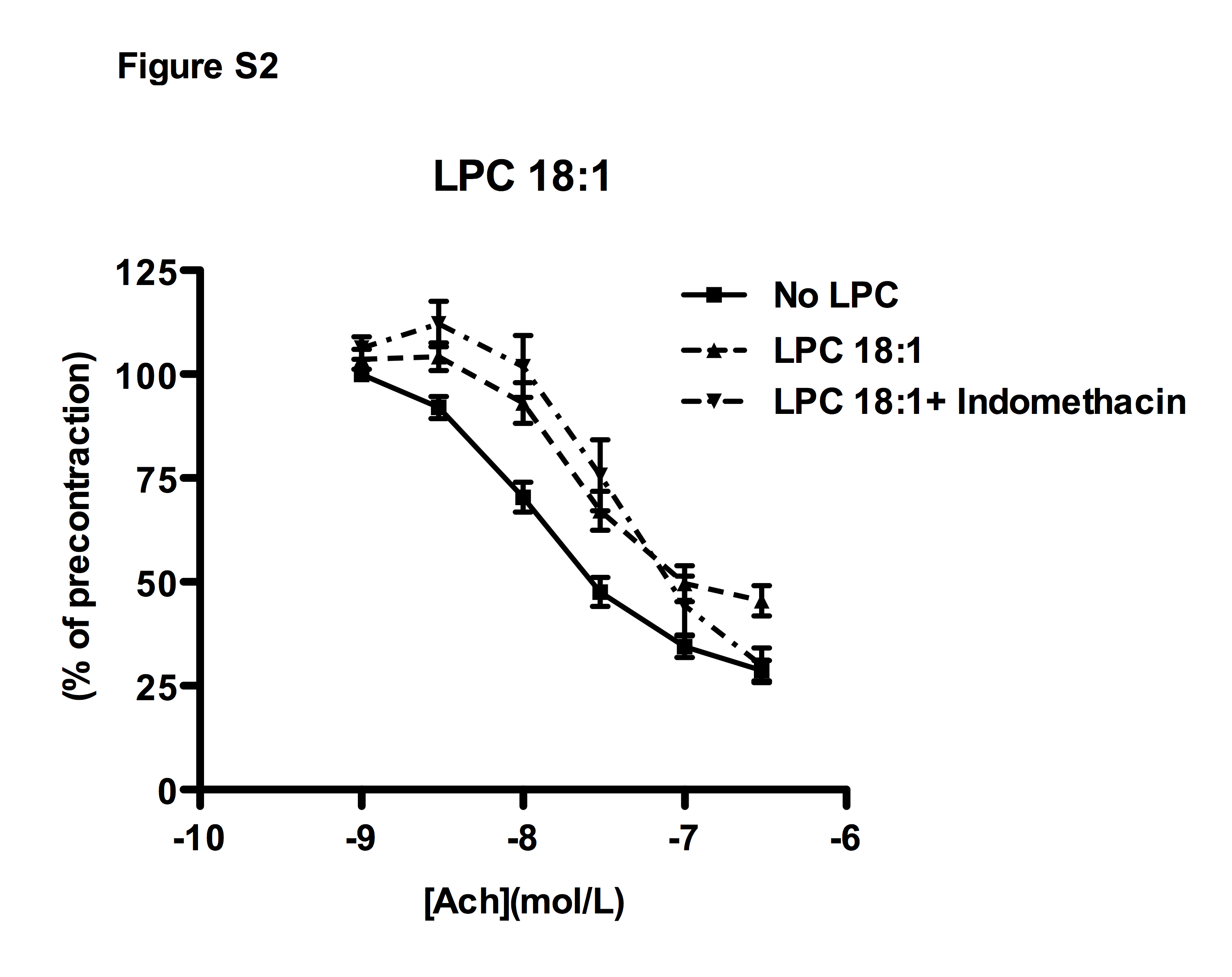

Supplement: Figure S2 — LPC 18:1-induced attenuation of relaxation is not affected by indomethacin. The rings were preincubated without (no LPC) or with 10 µM LPC 18:1 for 30 minutes, followed by precontraction with NE and cumulative addition of ACh. Relaxation values were expressed as a percentage of the NE-induced contraction. Results are mean ± SEM of 12 rings for each case from 6 mice. (TIFF) [file pone.0065155.s002.tiff]

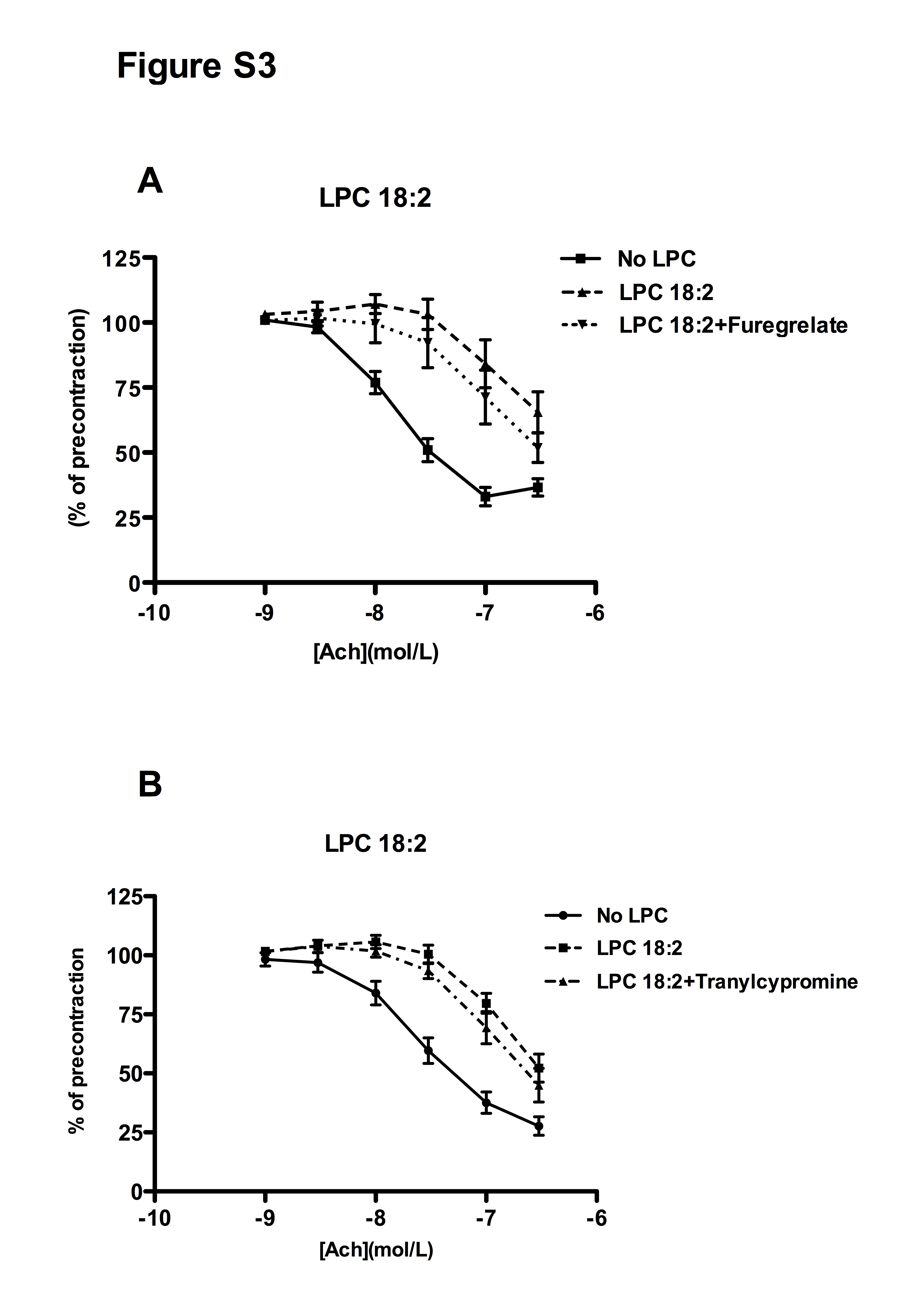

Supplement: Figure S3 — Furegrelate and tranylcypromine fail to recover relaxation attenuated by LPC 18:2. The rings were preincubated without (no LPC) or with LPC 18:2 in the absence or presence of 10 µM furegrelate (A) or 10 µM tranylcypromine (B) for 30 minutes, followed by precontraction with NE and cumulative addition of ACh. Relaxation values were expressed as a percentage of the NE-induced contraction. Results are mean ± SEM of 12 rings for each case from 6 mice. (TIFF) [file pone.0065155.s003.tiff]

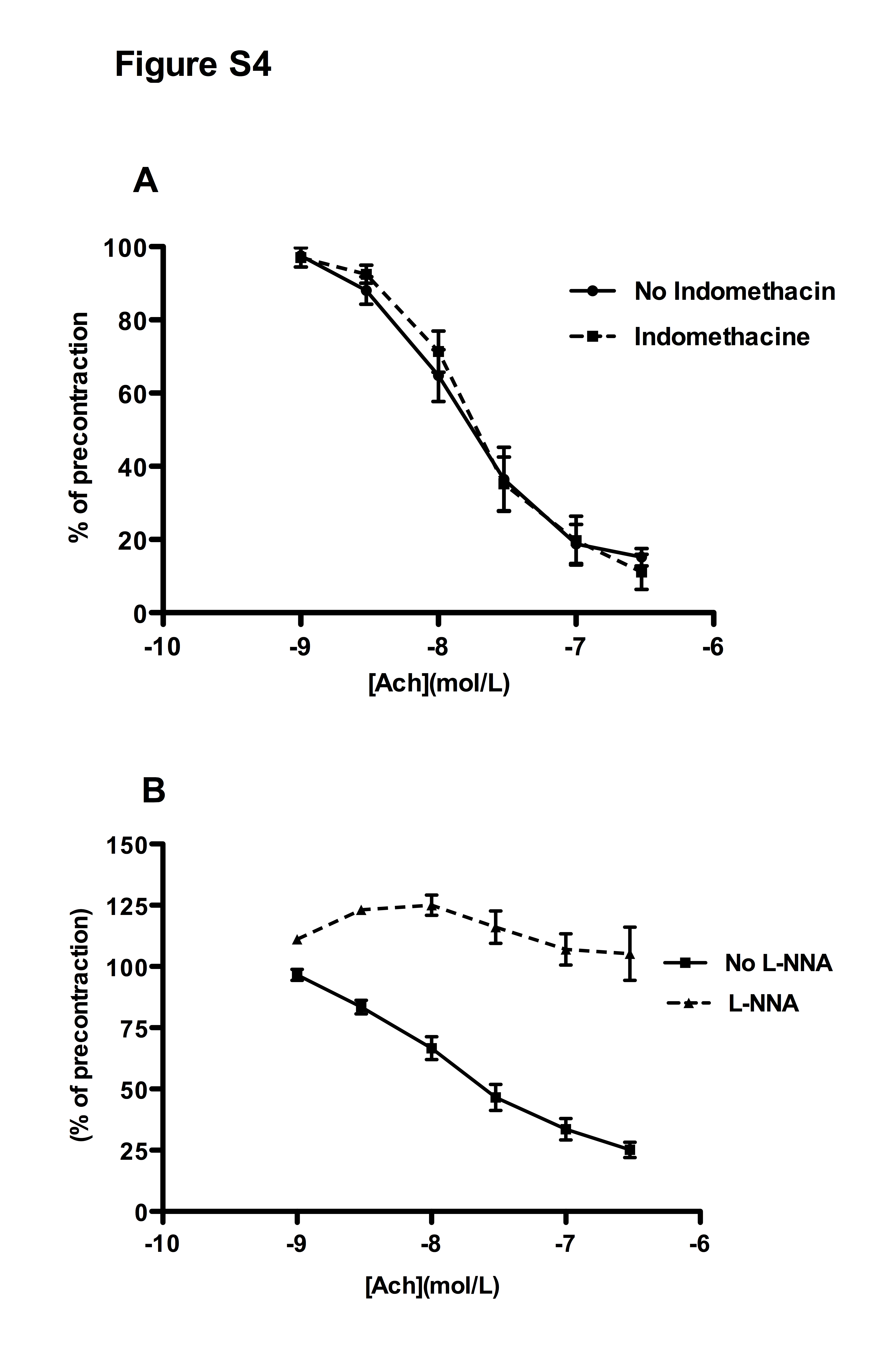

Supplement: Figure S4 — Inhibition of eNOS by L-NNA but not of COX by indomethacin abolishes Ach-induced relaxation in mouse aortic rings. The rings precontracted with NE were relaxed by a cumulative addition of Ach in the absence (no L-NNA) or presence of 200 µM L-NNA (A) or 20 µM indomethacin (B). Relaxation values were expressed as a percentage of the NE-induced contraction. Results are mean ± SEM of 8 rings for each case from 4 mice. (TIFF) [file pone.0065155.s004.tiff]

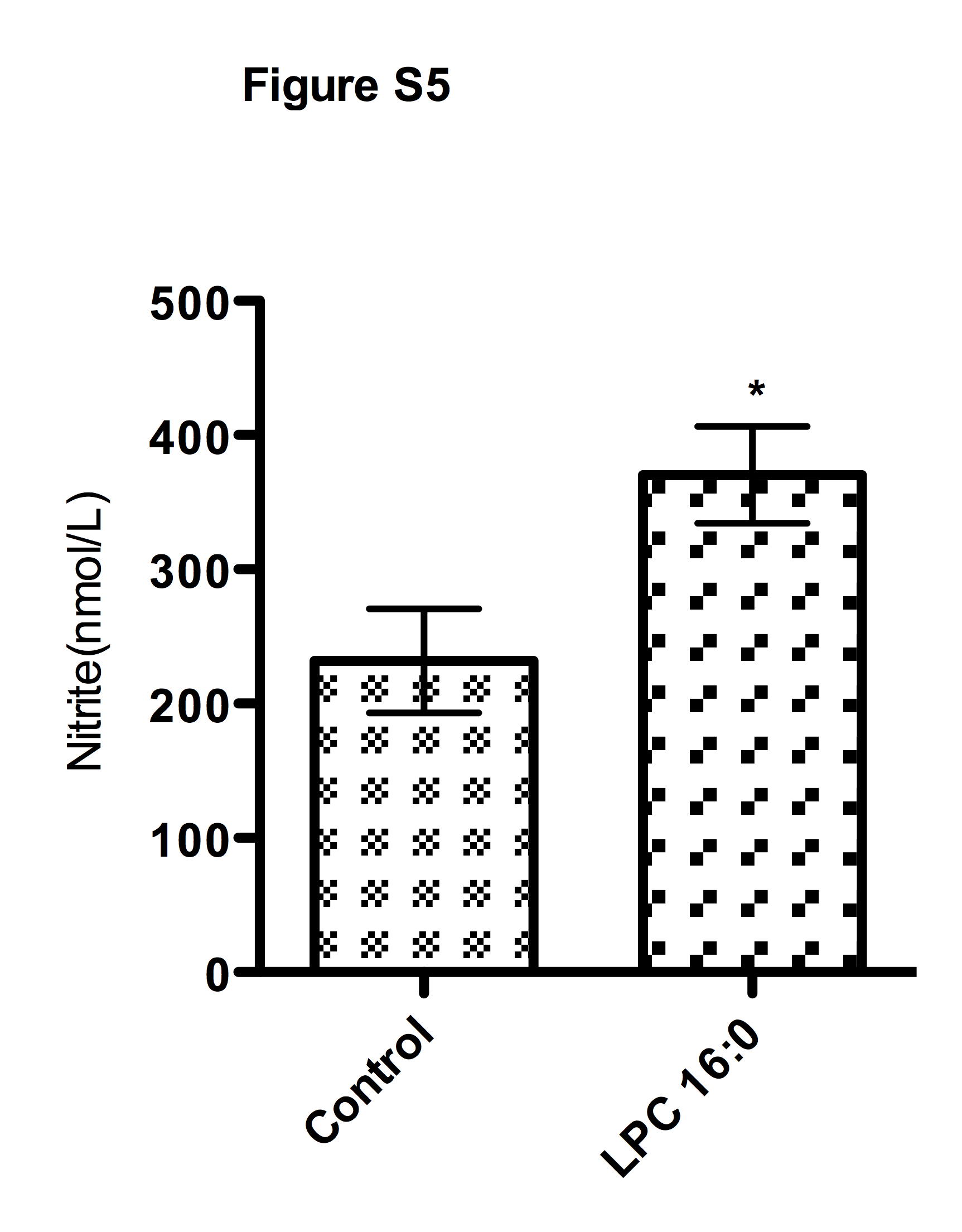

Supplement: Figure S5 — LPC 16:0 increases nitrite levels released from aortic rings exposed to Ach. The rings were precontracted with NE and relaxed by cumulative addition of Ach. After wash-out of ACh, the same rings were preincubated with 10 µM LPC 16:0 for 30 min followed by a new contraction-relaxation cycle. The nitrite levels were determined in incubation buffers after the first (control) and the second contraction-relaxation cycle (LPC 16:0). Results are mean ± SEM of 16 rings for each case from 4 mice. When LPC was omitted the nitrite levels released from rings were similar in the first and the second contraction-relaxation cycle (not shown). (TIFF) [file pone.0065155.s005.tiff]

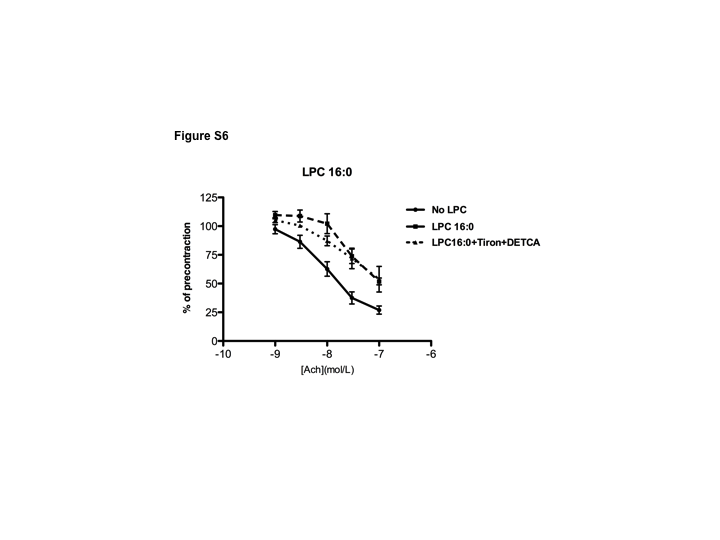

Supplement: Figure S6 — Combination of DETCA (SOD inhibitor) and TIRON (superoxide ion scavenger) fail to counteract LPC 16:0-induced attenuation of relaxation. The rings were preincubated without (no LPC) or with 10 µM LPC 16:0 in the absence or presence of DETCA (10 µM) and TIRON (100 µM) for 30 minutes, followed by precontraction with NE and cumulative addition of ACh. Results for each condition are mean ± SEM of 8 rings from 4 mice. (TIFF) [file pone.0065155.s006.tiff]
